# Circulating free mitochondrial DNA concentration and its association with erlotinib treatment in patients with adenocarcinoma of the lung

**DOI:** 10.3892/ol.2014.2006

**Published:** 2014-03-28

**Authors:** CHU-YUN HUANG, YUH-MIN CHEN, CHIEH-HUNG WU, CHUN-MING TSAI, YU-CHIN LEE, REURY-PERNG PERNG, JACQUELINE WHANG-PENG

**Affiliations:** 1 Department of Chest Medicine, Taipei Veterans General Hospital, School of Medicine, National Yang-Ming University, Taipei 112, Taiwan, R.O.C.; 2 Centre of Excellence Cancer Research, Taipei Medical University, Taipei 112, Taiwan, R.O.C.

**Keywords:** epidermal growth factor receptor, lung cancer, mitochondria, tyrosine kinase inhibitor

## Abstract

Changes in circulating free DNA concentrations have been correlated with chemotherapeutic effects in solid tumors. The present study was designed to determine and compare the changes in circulating free mitochondrial DNA (mtDNA) concentrations prior to and following erlotinib treatment, as well as the potential prognostic value of plasma mtDNA. Patients with adenocarcinoma of the lung who were to receive erlotinib treatment were enrolled in the present study once informed consent had been obtained. Patient plasma samples were collected immediately prior to starting erlotinib treatment, on days 15 and 29 following the initiation of erlotinib treatment and also when the patient’s disease had progressed. The most common erlotinib treatment response was a partial response (PR), achieved in 26 (49.1%) of the 53 enrolled patients, followed by stable disease (SD) in 13 patients (24.5%) and progressive disease (PD) in 14 patients (26.4%). Plasma mtDNA concentrations were significantly decreased on day 15 compared with day 0 in the patients with PD (P=0.028) or in those patients without a response to erlotinib treatment (SD and PD; P=0.007). Plasma mtDNA concentrations were similar or elevated on day 15 compared with day 0 in the patients with a PR (P=0.808). The concentration of plasma mtDNA did not correlate with progression-free survival (PFS). Tumor epidermal growth factor receptor (*EGFR*) mutation status (activating mutations in 16 patients and wild-type in 14 patients) did not correlate with the concentration of plasma mtDNA (P=0.951). Plasma mtDNA levels did not correlate with the PFS of the patients when they received erlotinib treatment. The plasma mtDNA levels were decreased on day 15 in those patients who had disease progression following erlotinib treatment. These results demonstrate that plasma mtDNA is of weak clinical utility as a screening, diagnostic or prognostic tool in lung cancer patients.

## Introduction

Lung cancer is the leading cause of cancer-associated mortality worldwide, and non-small cell lung cancer (NSCLC) accounts for >80% of all cases. In total, <20% of NSCLC patients are effectively cured of the disease due to the lack of effective early diagnostic methods, the high frequency of recurrence following curative surgery and the fact that >70% of patients are in the advanced stages of the disease at the time of diagnosis (1–3).

The presence of circulating free DNA in the plasma of patients with malignant neoplasms has been a well-established concept for >30 years. Studies have been undertaken to analyze the plasma circulating free DNA concentrations, together with the genetic and epigenetic alterations of the tumor DNA of patients who suffer from numerous types of cancer, including colon, breast, prostate and germ cell tumors (4–6). It has been previously demonstrated that patients suffering from malignant diseases have increased amounts of cell-free nucleic acids in their circulation; this circulating cell-free DNA is elevated in cancer patients and decreases in response to effective treatment (3,4,7).

As tumor tissues contain high copy numbers of mitochondrial DNA (mtDNA), the detection of plasma mtDNA may act as a sensitive tool for the identification of changes in disease status during cancer treatment. The present study investigated whether plasma mtDNA concentrations in lung cancer patients with adenocarcinoma were altered during treatment with erlotinib, an epidermal growth factor receptor-tyrosine kinase inhibitor (EGFR-TKI), and whether this change has the potential to be used as a predictor of treatment response.

## Materials and methods

### Patients

The present study procedure was approved by the Taipei Veterans General Hospital Institutional Review Board (VGHIRB no. 98–11–05). Patients with adenocarcinoma of the lung who were to receive erlotinib treatment were enrolled in the study once informed consent had been obtained. Blood samples were collected immediately prior to the administration of the first dose of erlotinib, on days 15 and 29 following the initiation of erlotinib treatment and also when the disease had progressed.

### Quantification of plasma free circulating mtDNA

Plasma was collected in EDTA tubes and centrifuged at 405 × g for 5 min. Each plasma sample (20 μl) was mixed with 20 μl of a preparation buffer that contained 2.5% Tween-20, 50 mM Tris and 1 mM EDTA. This mixture was digested with 16 μg proteinase K (Qiagen, Valencia, CA, USA) at 50°C for 40 min and diluted with 160 μl Tris-EDTA buffer after 5 min of heat deactivation at 95°C. Following centrifugation at 10,625 × g for 10 min, 1 μl of supernatant containing 0.1 μl of an equivalent quantity of the plasma was used as a template for quantitative PCR (qPCR) without purification. The copy number of mtDNA was measured by qPCR (TaqMan) performed on a LightCycler 1.2 using a LightCycler FastStart DNA Master Plus HybProbe kit (Roche Diagnostics, Mannheim, Germany). The primers and probe were obtained from PreVision Medical Corporation (New Taipei City, Taiwan, China) and selected to amplify 194-bp fragments in the mtDNA conserved region. The primer sequence used was AATTTCGTGCCAGCCACCGC and the probe sequence used was CTACGAAAGTGGCTTTAACA. Reactions were performed in a total volume in 20 μl, consisting of 4 μl 5× Master Mix (PreVision Medical Corporation), 0.8 μM probe and 0.4 μM of each primer. Thermocycling was performed in 20-μl glass capillary tubes. Following a 10 min activation step at 95°C, the reactions were subjected to 45 cycles of 95° for 15 sec, 60°C for 30 sec and 72°C for 5 sec. The absolute equivalent amount of the mtDNA copy number in each sample was determined by a standard curve with serial dilutions of the cloned plasmid.

### EGFR mutation analysis

*EGFR* mutation analysis was performed utilizing nucleotide sequence analysis. The VarientSEQr™ Resequencing Primer Set (Applied Biosystems, Foster City, CA, USA) was selected for mutational analysis of the tyrosine kinase domain, located at exons 18–21 of the *EGFR* gene. Genomic DNA was extracted from paraffin blocks, exons 18–21 were amplified and uncloned PCR fragments were sequenced and analyzed in the sense and antisense directions for the presence of heterozygous mutations. All the sequence variations were confirmed by multiple, independent PCR amplifications and repeated sequencing reactions. *EGFR* activating mutations were defined as those with exon 19 deletions or exon 21 L858R.

### Efficacy evaluation

A chest computed tomography scan (including the liver and adrenal glands) was performed within three weeks prior to the initiation of erlotinib treatment, at one and three months following the initiation of erlotinib treatment and then every three months thereafter. Treatment response evaluation was performed according to the Response Evaluation Criteria in Solid Tumors (RECIST) group criteria version 1.1 (8). Progression-free survival (PFS) was calculated from the date of starting erlotinib treatment to the earliest sign of disease progression, as determined by means of the RECIST criteria, or mortality from any cause. If disease progression had not occurred at the time of the last follow-up visit, PFS was considered to have been censored at that time.

### Statistical analysis

Survival curves were drawn using the Kaplan-Meier method. The log-rank test was used for comparisons of data. The plasma mtDNA levels in the patients who received erlotinib treatment were compared using the independent samples t-test, non-parametric Mann-Whitney test and the Wilcoxon test for different conditions. P<0.05 was considered to indicate a statistically significant difference. All statistical analyses were performed using SPSS software (SPSS, Inc., Chicago, IL, USA).

## Results

### Patient characteristics and treatment response

Plasma was prospectively collected from 53 patients, including 20 males and 33 females, with a mean age of 64 years and a range of 30–88 years. The performance status was 0 in four patients, 1 in 36 patients, 2 in nine patients and 3 in four patients. Of these 53 patients, the most common response to erlotinib treatment was a partial response (PR) in 26 patients (49.1%), followed by stable disease (SD) in 13 patients (24.5%) and progressive disease (PD) in 14 patients (26.4%). Of the 30 patients who had tumor tissue samples available for *EGFR* mutation analysis, 16 patients had *EGFR* activating mutations and 14 patients were *EGFR* wild-type.

### Plasma mtDNA concentration correlates with erlotinib treatment response

**Figure 1:**
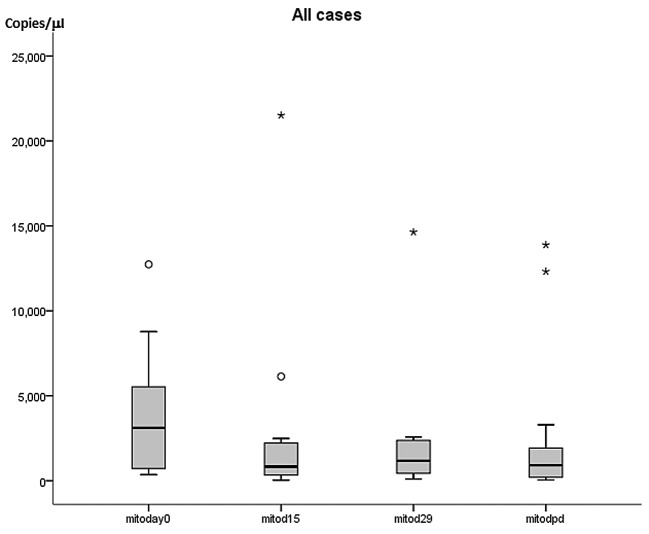
Plasma mtDNA concentration of the patients treated with erlotinib on days 0, 15, 29 and at the time of disease progression (dpd). ∘Minor outliers; ^*^extreme outliers; mtDNA, mitochondrial DNA.

In the patients with a PR to erlotinib treatment, the mean plasma mtDNA concentration was 2,907±757 copies/μl (mean ± standard error of the mean) on day 0 (n=26), 3,109±986 copies/μl on day 15 (n=23), 2,077±473 copies/μl on day 29 (n=24) and 808±446 copies/μl with disease progression (n=7). In the patients with PD following erlotinib treatment, the mean plasma mtDNA concentration was 3,776±1,014 copies/μl on day 0 (n=14), 1,567±387 copies/μl on day 15 (n=13), 3,159±1,667 copies/μl on day 29 (n=8) and 6,387±2,189 copies/μl with disease progression (n=9) (Table I; Fig. 1).

**Figure 2:**
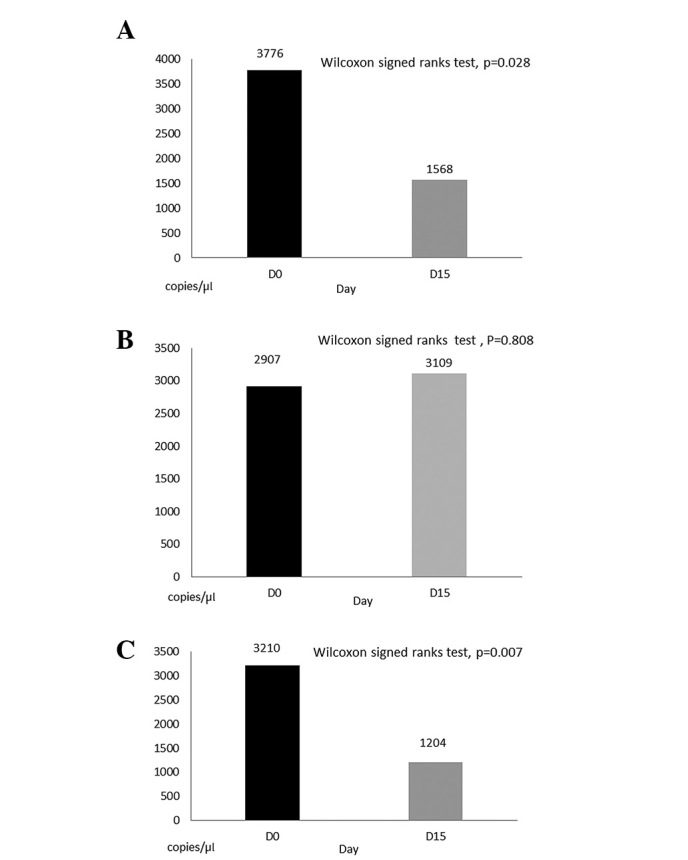
Changes in plasma mtDNA levels during erlotinib treatment. (A) Plasma mtDNA concentrations were significantly decreased on day 15 compared with day 0 in patients with PD following erlotinib treatment (Wilcoxon signed-rank test, P=0.028). (B) Plasma mtDNA concentrations were significantly decreased on day 15 compared with day 0 in patients with no response (SD+PD) to erlotinib treatment (Wilcoxon signed-rank test, P=0.007). (C) Plasma mtDNA concentrations were either decreased, similar or elevated on day 15 compared with day 0 in patients with a PR to erlotinib treatment (Wilcoxon signed-rank test, P=0.808). mtDNA, mitochondrial DNA; PD, progressive disease; SD, stable disease.

**Figure 3:**
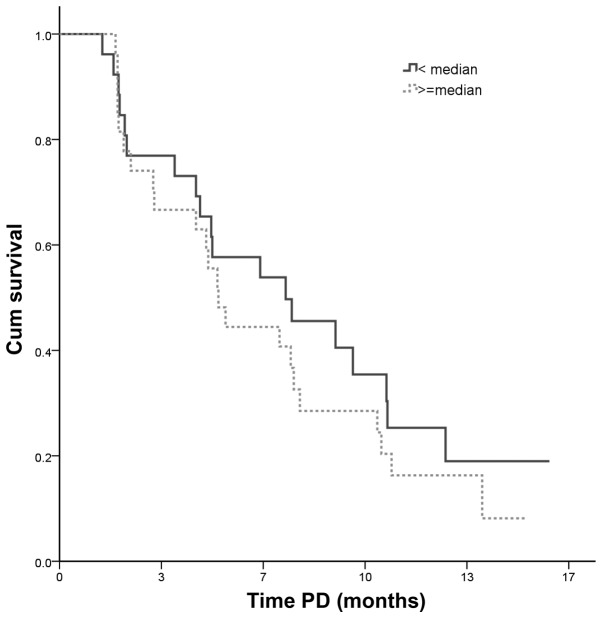
PFS following the initiation of erlotinib treatment. Patients with a plasma free mitochondrial DNA concentration lower than median value had a longer PFS time compared with patients with plasma free mtDNA concentration that was higher than the median value, however, this difference was not statistically significant (low free mtDNA value: n=26, censor 7, median 7.4 months; high free mtDNA value: n=27, censor 4, median 5.2 months; P=0.367). PFS, progression-free survival; mtDNA, mitochondrial DNA; PD, progressive disease; Cum, cumulative.

Sequential changes in plasma mtDNA concentration in individual patients were significantly decreased on day 15 compared with the levels observed on day 0 in the patients with PD following erlotinib treatment (Wilcoxon signed-rank test, P=0.028; Fig. 2A) or with no response (SD + PD) to erlotinib treatment (Wilcoxon signed-rank test, P=0.007; Fig. 2B). Plasma mtDNA concentrations in patients with a PR to erlotinib treatment did not differ significantly on day 15 compared with day 0 (Wilcoxon signed-rank test, P=0.808; Fig. 2C), as the levels of individual patients varied, with decreases, increases or similar concentrations recorded. There was no statistically significant difference in plasma mtDNA concentrations between days 0 and 29 or the day of disease progression in individual patients with a response, SD, PD or no response to treatment. When the patients were divided into a PR group and a non-PR group, the PR group had significantly higher plasma mtDNA concentrations than the non-PR group on day 15 and when the disease progressed (independent samples t-test, P=0.004 and P=0.003, respectively). Although the patients with low plasma mtDNA levels had a marginally longer PFS, there was no statistically significant difference in PFS between the patients with high or low plasma mtDNA concentrations (P=0.367, Fig. 3). There was no significant correlation of the plasma mtDNA levels with tumor *EGFR* wild-type or activating mutations (P=0.951).

## Discussion

A number of serum or plasma markers, including cytokeratin fragment 21–1, carcinoembryonic antigen, carbohydrate antigen 19–9 and squamous cell carcinoma antigen, have been considered as potential aids in the screening or early detection of lung cancer. The levels of these circulating markers have been reported to be elevated in certain lung cancer patients (9–11). However, not all cancer patients had elevated markers.

Circulating free DNA has been found in healthy individuals, patients with non-malignant diseases and in those with various malignancies, including lung cancer (2,3,12–19). However, several studies have revealed that these circulating free DNA concentrations are notably higher in individuals with various malignancies than in healthy controls (2,16,20). As a result, numerous mechanisms have been hypothesized to explain the release of circulating free DNA into the circulation by tumor-host or tumor-virus interactions (16,17).

The detection of plasma circulating tumor DNA in lung cancer patients may be a potential marker for screening, diagnosis, prognosis, monitoring of treatment response and the early detection of disease progression. The best examples are circulating *EGFR* mutation markers, which may be used to gauge response, monitor progression and possibly detect newly acquired mutations (21,22). Circulating mtDNA is a type of circulating free DNA, the study of which has been limited in comparison. The present study attempted to identify whether this circulating marker may be used as a predictor in EGFR-TKI treatment for patients with pulmonary adenocarcinoma.

The detection of specific mitochondrial mutations in a patient’s plasma would be a useful measurement to investigate novel diagnostic tools for lung cancer. However, the specific mitochondrial mutations that occur in lung cancer have rarely been studied, nor have they been reported in plasma samples, although mutations in the mitochondrial genome have been documented for a number of other types of cancer (23,24). Despite this, the detection of free mtDNA in plasma is as simple to perform as the detection of plasma free DNA in cancer patients.

In previous studies, lung cancer patients who had received chemotherapy were reported to have significantly increased plasma free DNA concentrations at the time of disease progression (20,25,26). In the present study, patients with a response to erlotinib treatment had significantly higher plasma mtDNA concentrations than the remaining patients (the no response group) on day 15 following erlotinib treatment and when their disease progressed (P=0.004 and P=0.003, respectively). The initial elevation of mtDNA on day 15 could be explained by the erlotinib-induced destruction of tumor cells, which may have been accompanied by the release of mtDNA into the circulation. Elevation of mtDNA in patients who progressed following an initial response may be due to the increased proliferation and turnover of these tumor cells. By contrast, mtDNA levels were lower on day 15 in those patients resistant to erlotinib treatment. This may be due to the lack of a tumoricidal effect of erlotinib treatment on these tumor cells, or more likely due to a reduced tumor cell turnover rate in the first two weeks of erlotinib treatment in these patients.

The present study demonstrated that plasma mtDNA levels did not correlate with PFS when pulmonary adenocarcinoma patients received erlotinib treatment, and that there was also no association with tumor *EGFR* mutation status. To the best of our knowledge, these findings have not been reported in any previous studies. According to these results, plasma mtDNA appeared to be of weak clinical utility as a screening, diagnostic or prognostic tool in lung cancer patients.

## Figures and Tables

**Table I:** Plasma free mtDNA levels in 53 erlotinib-treated patients.

Parameter	Partial response	Stable disease	Progressive disease
Day 0			
No. of patients	26	13	14
mtDNA concentration, copies/μl			
Mean ± SEM	2907±757	2601±874	3776±1,013
Range	154–17,510	228–10,644	63–12,733
Day 15			
No. of patients	23	11	13
mtDNA concentration, copies/μl			
Mean ± SEM	3109±986	774±221	1568±387
Range	26–21,511	60–2,395	54–4,925
Day 29			
No. of patients	24	11	8
mtDNA concentration, copies/μl			
Mean ± SEM	2077±473	2935±948	3159±1,667
Range	56–7,587	107–9,383	390–14,637

mtDNA, mitochondrial DNA; SEM, standard error of the mean.
